# Using signalling theory to assess the Government of Ghana’s risk communication during the COVID-19 pandemic

**DOI:** 10.4102/hsag.v27i0.1623

**Published:** 2022-07-27

**Authors:** Martin Owusu Ansah, Lucy Afeafa Ry-Kottoh, Enya B. Ameza-Xemalordzo, Godfred Aawaar

**Affiliations:** 1 Department of Marketing and Corporate Strategy, College of Humanities and Social Sciences, Kwame Nkrumah University of Science and Technology, Kumasi, Ghana; 2 Department of Publishing Studies, College of Art and Built Environment, Kwame Nkrumah University of Science and Technology, Kumasi, Ghana; 3 Department of Accounting and Finance, College of Humanities and Social Sciences, Kwame Nkrumah University of Science and Technology, Kumasi, Ghana

**Keywords:** COVID-19, coronavirus, pandemic, communication, partial least square modelling

## Abstract

**Background:**

The proliferation of information through social media and on other communication networks during the corona virus disease 2019 (COVID-19) era altered information transfer in many countries. The content of the messages from government officials, media coverage and alternative narratives, affected the level of compliance in adhering to the various health protocols amongst the public.

**Aim:**

This article aimed to determine the relationship between the message used, media coverage, alternative narratives, the public’s attitude towards staying at home and their commitment to stay at home during the COVID-19 pandemic campaign period in Ghana.

**Setting:**

A total of 352 respondents was sampled from the Kumasi metropolis.

**Methods:**

A survey sample strategy and a convenience sampling technique were used while structural equation modelling with Partial least square (PLS) version 3.0 was used for the analysis.

**Results:**

The study revealed that the nature of media coverage employed and the alternative narratives had a significant positive effect on the attitude of the respondents, whilst the content of the message had insignificant effects on the attitude of the public. Finally, the attitude of the people had a significant positive influence on their respective commitment to stay home.

**Conclusion:**

Developing countries in Africa need to fight pandemics using purely subsidised health officials or directorates rather than have government-appointed health experts and officials spearheading activities during a pandemic.

**Contribution:**

This study contributes to the clear understanding on some COVID-19 misinformation, the distinctive cost it poses to public health delivery in developing economies and the potential strategy of using neutral public health persons to curb the spread of the virus.

## Introduction

The spread of the coronavirus continues to receive media attention, raising security concerns, affecting healthcare and even the teams gathered by governments to control its spread (Shesgreen 2020). The virus became a worldwide health epidemic, infecting and killing people across the world, with numbers growing across the continents. Health officials across the world were overwhelmed by the increasing occurrence and spread of the outbreak (Ram 2020). According to Kunreuther and Slovic (2020) and Jones (2020), the challenges associated with coronavirus disease 2019 (COVID-19) and the virus control were growing daily. Because of that, many organisations overhauled their clinical rotations (Comer et al. 2020) in the affected and yet-to-be affected countries. The pandemic forced the parameters of market-based economies, health structures and a swamping of utmost egalitarian societies across the globe (Grint 2020) to devise strategies in curbing the spread of the virus. Zhang and Chen (2020) observed that governments regularly readjusted their intervention techniques in curbing the spread of the pandemic through several approaches, such as airport closures, border closures, lockdowns, public gathering prohibition and so on. Similarly, Lamptey and Serwaa (2020) observed that the rise of the novel coronavirus increased insurgency in the healthcare industry.

Ghana recorded its first case on 12 March 2020 after two people tested positive on their return to Ghana from Norway and Turkey. As of 12 May 2020, at 08:31 h GMT, Ghana had recorded 4700 cases, with 494 recoveries and 22 deaths (Worldometer 2020). Given the spread, the Government of Ghana instituted measures and presented frequent updates on the pandemic as part of the campaign strategy employed to curb the spread of the virus. These measures included a 14-day mandatory quarantine imposed on persons who arrived in the country before the closure of the country’s borders, periodic televised addresses by the President of the Republic of Ghana, periodic updates on the cases by the Ministry of Information, publicity by the frontline hospital staff or experts from the Ministry of Health, establishing coronavirus hotline telephone numbers, introducing the GH COVID-19 Tracker App and enforcing the restriction of movement in an attempt to control the spread of the virus. In spite of that, many of these preventive measures were flouted by people leading to the spread of the virus.

According to Abdulai et al. (2021), assessing the right health information was a test for many people in Ghana, who did not have the medical knowledge to access and comprehend the information available on many platforms. Studies have shown that factors such as political gimmicks during the COVID-19 campaigns by opposition parties, unstructured media coverage on the fight (Wen et al. 2020), inadequate message structure on COVID-19, unscrupulous business advantage during the COVID-19 period and inadequate health professionals, all deluded people not to stay home to minimise the spread. Overy (2020) observed that some people used the COVID-19 period to carry out vendettas against their opponents as one factor that affected the whole campaign structure in Ghana. It is always challenging when governments mediate in emergencies, such as the coronavirus pandemic. Stories are adjusted by many recipients to frame the organisers of the campaign in ways that fit a political storyline in most emergencies (Bennett & Perez 2020; Terry 1997). Also, excessive blaming of people in emergencies makes it tough to separate actions from discourse and that causes harm to public awareness (Luton 1994; McSwite 2005; Patterson 2001). Likewise, receivers of messages usually attach weights to the signals based on preconceived notions of the signaller (Branzei et al. 2004; Ehrhart & Ziegert 2005).

Studies by Zhang and Wiersema (2009) and Basdeo et al. (2006) investigated the effectiveness of signals sent by firms. Goranova et al. (2007) and Carter (2006) analysed the effect of signals on individuals or managers whilst Chung and Kalnins (2001) investigated on the effect of product signals, with Miller and Triana (2009) focusing on directors’ ‘message signal–receiver effect’. According to Gammoh, Voss and Chakraborty (2006), the use of the ‘signal–receiver’ relationship has been common in consumer research and limited in other areas of study. An inference could therefore be drawn on scant research on the use of signals in creating awareness about a pandemic, such as the COVID-19. Commentaries from politicians, religious leaders, celebrities, health experts and self-acclaimed traditionalists all contributed to the possibility of sending mixed messages to the public. People were tired of the blameworthiness, fabrications and political oratory and so disconnected from the reality of the coronavirus spread (Zavattaro & McCandless 2020). Ram (2020) investigated the ‘Coronavirus Research Trends’ using a ‘50-Year Bibliometric Assessment’ with productive countries, productive institutes, productive authors, productive journals, characteristics of highly cited papers, author keyword analysis and hot research areas that were found from the African continent.

Besides the studies in epidemiology, clinical characteristics, treatment and clinical outcomes of confirmed cases, there are limited studies on the human infection of COVID-19 (Addo et al. 2020; Cortegiani et al. 2020; Li et al. 2020). According to Sibiri, Zankawah and Prah (2020), although developed countries received more attention because of their scientific innovativeness in their quest to contain the spread of the virus, African countries received little attention because their innovations were few although significant. Whilst several studies have been carried out on COVID-19 in Asia, Australia, North America, South America and Europe, there is little research on communication or messages used during the pandemic.

In this study, the authors quantitatively reviewed the content of messages during the height of Ghana’s COVID-19 period, the media coverage on the pandemic and how alternative narratives impacted people’s commitment to stay at home. We examined the association between messages used and attitudes of people towards staying at home during the pandemic period, investigated the relationship between media coverage of COVID-19 and attitudes of people, established the relationship between alternative narratives on COVID-19 and attitudes of people and analysed the relationship between attitudes of people and their commitment to stay home during the COVID-19 period.

## Literature

### Signalling theory

Signalling theory (ST) propounded by Spence (1973) explains how a signaller transports substantial information about his or her activities as in products or services to a recipient or receiver. The theory is appropriate for defining behaviour when two parties have access to varied types of information (Connelly et al. 2011a; Connelly, Ketchen & Slater 2011b). The signallers are insiders, officials and administrators who obtain information about a person (Spence 1973). According to Kirmani and Rao (2000), signals could be sent on a product as well as on organisations (Ross 1977) that are not accessible to outsiders. Signalling, therefore, takes place within or between organisations (Lester et al. 2006; Rynes, Bretz & Gerhart 1991). As the pandemic period became sensitive, and politicians, celebrities, opinion leaders and reference leaders expressed diverse views on government communication, environmental distortions created the propensity to reduce the observability of the signal. In such a situation, people were likely to doubt the information provided by the signaller. According to Carter (2006), the moment the media reports on press releases in an environment where ideas are diverse, potential distortions are likely to be introduced. Branzei et al. (2004) also observed that external referents could change the information unevenness. Earlier research demonstrated that signals must be noticeable to the recipient and the level at which signalling is obvious depends on how attentive the receiver is in scanning the environment for indications (Bruton et al. 2009; Connelly et al. 2011; Janney & Folta 2006).

### Message, media coverage and attitude

A message is a discrete constituent of communication proposed by the source for consumption of the receiver or a group of receivers (Salloum et al. 2018). According to Vasterman, Yzermans and Dirkzwager (2005) and Young et al. (2013), a message shrouded in an unbalanced reporting of healthcare crises has a likelihood to create a split in ‘actual versus perceived’ risks, leading to under- and overreaction to decisions. A well-crafted message on COVID-19 will influence people’s attitude to either accept or reject an idea based on instructions from health professionals. Media coverage refers to all blog articles, video contents or other types of digital content that are produced by individuals or organisations to attract attention, interest, desire and be received by their target audience (Brossard 2013). The nature of media coverage highly influences people’s feelings and attitudes whenever there is an outbreak of transmittable diseases (Mairal 2011; Young et al. 2013). As a result, news associated with infectious diseases has a greater likelihood of causing alarm and influencing people’s feelings (Tetlock 2007; Wen et al. 2020). In the current study, the signaller is the Government of Ghana providing information through its Ministries of Health and Information to the people of Ghana who are the receivers. Any inconsistency between the signaller and the message being sent can lead to poor signalling, which can distort the information and influence compliance. Also, people’s disposition towards the carriers of the message is likely to positively or negatively influence the way message is received.

Attitude is a significant internal influencer, affecting people’s decision processes (Abou-Youssef et al. 2020), and ‘a learned predisposition to behave in a consistently favorable or unfavorable way with respect to a given object’ (Kanuk & Schiffman 2000:253). Attitude has an influence on people’s thoughts towards an object (Fazio 1986) and is a motivational factor that drives them towards an action or hinders a certain behaviour (Kanuk & Schiffman 2000). In this study, it was observed that people’s attitudes during the COVID-19 period (whether positive or negative) were likely to influence their behaviour to act favourably or otherwise towards the guidelines they were expected to observe.

### Risk communication and fear appeals

‘Risk communication’ refers to the idea of managing risk elements and risk consequences (Heath 1995) or as a consolidative procedure of exchanging information and views amongst diverse stakeholders from the individual level to the organisational level (National Research Council 1989). Studies by Reynolds and Seeger (2005), Roeser (2012) and Lim and Lee (2016) have observed that public health communication that relies on individual sentiments, particularly fear based or undesirable emotions, within a risk communication system is likely to affect changes in citizens’ cooperative behaviours with respect to an envisioned outcome. It then demonstrates that the nature of a particular communication strategy has a greater effect on people’s tendency to behave favourably or unfavourably.

### Health policy campaigns and inadequate health officials

Policies and support procedures were instituted to create awareness within the COVID-19 period. Although introducing health officials to the public in the pandemic period was laudable, the use of frontline workers or health experts who were mostly government appointees in the campaign process created an aversion for people not to comply with most of the directives. As Bennett and Perez (2020) observed, it is challenging when the government mediates in emergencies, such as pandemics. Some members of parliament from the major political parties also politicised the campaign by posting their pictures on *veronica buckets* and tanks that were provided for hand washing. Other politicians also alleged that their constituents were not supplied with some treats or handouts because they were not members of a particular political party. The back-and-forth of ideological views about the campaign process affected the assimilation of information during the pandemic. That notwithstanding, it would have been expected that people would realise that the virus infected and killed many people irrespective of their political affiliations. Leaders have to be more pre-emptive, sensitive and recognise the effects of their short-term engagement (Gardiner 2020).

The lack of adequate health officials, such as public health directors, public health nurses and community health nurses, who could have salvaged the situation by providing strong case signals about the pandemic also contributed to the attitude of the people in compliance. Some comments culled from responses of some open-ended questions in the questionnaires indicated that:

• The campaign should have been carried out by neutral health officials, other than the communication minister.

• The pandemic should have been handled by the health officials in the country.

• Involving public health officers and nurses would have had a greater effect than the government controlling everything

• Ghana has become too political, so the involvement of health officials from the various community institutions would have been more appropriate than the government.

Health-related campaigns were more likely to be positive when they were drafted and carried out by the health officials instead of the government. Using health officials in the pandemic campaigns, to a large extent, reduces doubt and misinformation as the external nature of the threat of COVID-19 and its dynamics can make the ultimate impact unknown to government officials or politicians.

### Hypothesis statements

The study’s hypotheses were presented as follows:

**H1**: The message on the COVID-19 has a significant positive effect on the attitude of people.

**H2**: The media coverage on the COVID-19 has a significant positive effect on the attitude of people.

**H3**: The alternative narrative on the COVID-19 has a significant positive effect on the attitude of people.

**H4**: The attitude of the people has a significant positive effect on people’s commitment to stay home within the COVID-19 period.

## Methodology

### Sample and data collection

This study employed a survey strategy in collecting data from respondents during the COVID-19 period. The population comprised the entire people in the Kumasi metropolis; the exact sampling frame could not be determined, whilst the convenience sampling technique was employed in the month of May 2020 for data collection. A total of 364 respondents were sampled from 20 April 2020 (a day after the 3-week lockdown imposed by the Government of Ghana) to 10 May 2020 and 352 participants responded willingly. Data collection was mainly done within the Kumasi metropolis, adhering strictly to the physical or social distance order that was in place. A combination of self-administered questionnaire and interviewer-administered questionnaire was used in the entire data collection process. Convenience sampling technique was used in eliciting the views of the respondents because there was no specific sampling frame. Data collection was carried out until the required sample – subject to related studies – was gathered for the analysis. The measurement items such as the ‘message’, ‘media coverage’, ‘alternative narratives’, ‘attitude’ and ‘commitment to stay home’ on the questionnaire were organised on a five-point Likert scale, with 1 representing ‘strongly agree’ and 5 ‘strongly disagree’. The questionnaire had two sections: information on participants in Section A, and all the variables (messages, media coverage, alternative narratives, attitude and commitment to stay home) in Section B (see Appendix 1). The items for the variables were developed from academic literature based on previous and current pandemic works.

In minimising the likelihood of probable bias resulting from a response, ‘N/A’ was included in each question as an alternative to guarantee the soundness of the questionnaire because it was a newly developed questionnaire. Two experts from the Kwame Nkrumah University of Science and Technology (KNUST) School of Business in Ghana and the Department of Marketing at the University of the Witwatersrand, South Africa, evaluated the measurement items by looking at the structure and phraseology of the instrument. Their recommendations were then used to update the questionnaire and tested on 10 people from the Kumasi metropolis – specifically, Oforikrom and Anwomaso communities. The comments from the pilot testing enhanced the adequacy of the content and wording of the instrument. Thus, the measurement item was seen to be dependable. As English is the indorsed language in Ghana, many of the participants could express themselves in the language, therefore making the data collection easier. However, those who could not write also participated as the research assistants were trained to translate it into the ‘Akan’ language – which many understood and which was also used in the collection process (Brislin 1986).

### Analysis

The analysis was carried out in two stages. Firstly, exploratory factor analysis (EFA) was used to validate the number of measurement items. Exploratory factor analysis was conducted using IBM SPSS version 24.0. The hypothesised relationship of the model was carried using structural equation modelling (SEM) via partial least square (PLS) version 3.0.

### Test on unidimensionality

A dimensionality assessment was carried out in testing the study’s sampling adequacy using the EFA. The Kaiser–Meyer–Olkin (KMO) of sampling adequacy was greater than 0.700 and Bartlett’s test of sphericity was significant (*p* < 0.05), indicating the variables were suitable for factor analysis (Meyer & Collier 2001; Pallant 2010:187). A principal component analysis with varimax rotation was used to extract appropriate factors for the study’s analysis. Significant factors were determined using the criterion of an Eigenvalue greater than 1 for factor loadings (Pallant 2010:192). Eigenvalues for the study’s constructs were message content on the COVID-19 (2.782), media coverage on the COVID-19 (2.601), alternative narratives on the COVID-19 (2.348), attitude (2.671) and commitment to stay home (2.822). The cumulative variance for the five factors was 71.0% (see Table 1).

**TABLE 1:** Dimensionality indicators of the exploratory factor analysis.

Constructs (number of items retained)	Factor loadings dimensionality model (EFA)
Message content on COVID-19 (4/5)	0.722; 0.751; 0.635; 0.717
Media coverage on COVID-19 (3/5)	0.856; 0.827; 0.704
Alternative narratives on COVID-19 (4/5)	0.876; 0.815; 0.723; 0.719
Attitude towards COVID-19 (3/5)	0.879; 0.843; 0.737
Commitment to stay home (3/5)	0.822; 0.747; 0.711

EFA, exploratory factor analysis.

Table 1 explains the number of items that were retained and rejected. The respective numerators and the denominators indicate the total and the numbers that were used for the study. The study used five items for each of the measurement variables, out of which some were not used because they loaded less than 50%. The retained values are shown in the factor loading section, with the ‘message content on COVID-19’ scoring 4 of the 5 questions (4/5), ‘media coverage on COVID-19’, 3 of the 5 questions (3/5), ‘alternative narratives on COVID-19’, 4 of the 5 questions (4/5), ‘attitude towards COVID-19’, 3 of the 5 questions (3/5) whilst ‘commitment to stay home’, 3 of 5 questions (3/5).

### Common bias assessment

Data were collected through a survey using questionnaires. Succeeding the recommendations of Podsakoff et al. (2003), several steps were taken to lessen the prospective risk of common method bias from the use of a single respondent. Firstly, the authors and the research assistants ensured informants were normatively committed by reassuring them of confidentiality and anonymity. Secondly, the measurement items were judiciously constructed to avoid any possible uncertainties.

### Partial least squares-structural equation modelling

Factors derived from the EFA were subjected to structural equation modelling using Partial least squares (PLS)’s version 3.0 of PLS Graph (Chin 2001). Partial least squares takes a component-based approach to the method of assessing the measurement model for the construct validation whilst using the structural model to determine the relationships of the stated hypotheses (Bock et al. 2005; Chin, Marcolin & Newsted 2003).

### Reliability and validity

Reliability was measured using internal consistency and composite reliability. Internal consistency was measured by Cronbach’s alpha values using IBM SPSS and composite reliability with the partial least squares-structural equation modelling (PLS-SEM). Reliability is demonstrated through high Cronbach’s alpha values ranging from 0.701 to 0.846 and composite reliability values ranging from 0.813 to 0.906 (Table 2). According to Nunnally (1978), a value of 0.7 as a standard for ‘modest’ reliability is appropriate in assessing composite reliability usefulness. All tested indicator loadings exceeded the recommended yardstick of 0.7 indicator reliability for all first-order dimensions (Chin 2010). Average variance extracted (AVE) values were used to show convergent validity. As indicated by Zott and Amit (2008), Croteau and Bergeron (2001) and Fornell and Larcker (1981), an AVE value ought to exceed 0.5 to ensure the convergent validity of the items. Also, the application of the exploratory principal component analysis is employed as part of the process of validation (Wilson 2014), demonstrating factor validity.

**TABLE 2:** Measure validation.

Measurement items	Cronbach’s alpha (∞)	Rho_A	Composite reliability	Average variance extracted	Factor loadings
**Alternative narratives (AN)**	0.702	0.730	0.813	0.522	0.634
AN1	-	-	-	-	0.789
AN2	-	-	-	-	0.782
AN3	-	-	-	-	0.673
AN4	-	-	-	-	-
**Commitment to stay home (C)**	0.846	0.863	0.906	0.764	0.914
C1	-	-	-	-	0.837
C3	-	-	-	-	0.869
C4	-	-	-	-	-
**Attitudes (AT)**	0.792	0.733	0.800	0.623	0.764
AT2	-	-	-	-	0.746
AT3	-	-	-	-	0.834
AT4	-	-	-	-	-
**Message (MIC)**	0.701	0.700	0.767	0.598	0.574
MIC3	-	-	-	-	0.971
MIC4	-	-	-	-	-
**Media coverage (MV)**	0.704	0.722	0.867	0.634	0.808
MV3	-	-	-	-	0.736
MV2	-	-	-	-	-

Note: AN, alternative narratives; AT, attitude; C, commitment to stay home; MIC, message; MV, media coverage.

### Discriminant validity

Discriminant validity measures the scope at which concealed factors are distinctive and should not relate so greatly that they seem to measure the same fundamental dimension (Siekpe 2005). Discriminant validity is recognised for assessment if the AVE values of a variable are larger than the squared correlation coefficients between variables (Barclay, Higgins & Thompson 1995; Fornell & Larcker 1981). In the current study, the squared correlations of the corresponding pairs of constructs were lower than the separate AVEs, indicating that the measurement model had good discriminant validity (Hair et al. 2014; Preacher & Hayes 2004). The results in Table 3 show a strong discriminant validity.

**TABLE 3:** Inter-construct correlation matrix.

Constructs	AN	AT	C	MIC	MV
Alternative narratives (AN)	1.000	-	-	-	-
Attitudes (AT)	0.483	1.000	-	-	-
Commitment to stay home (C)	0.409	0.762	1.000	-	-
Message (MIC)	0.411	0.447	0.442	1.000	-
Media coverage (MV)	0502	0.301	0.305	0.235	1.000

### Structural model of the relationships

**FIGURE 1:**
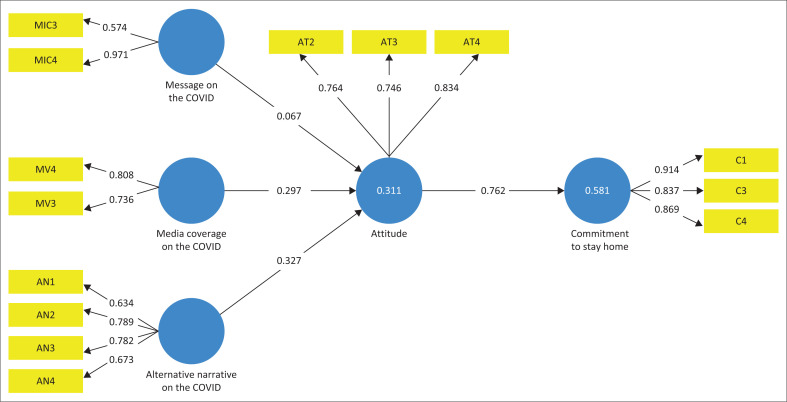
Structural equation model.

Results for the structural model used to examine the proposed hypotheses are shown in Table 4 and Figure 1. The study examined the impact of the independent variables: message, media coverage and alternative narratives on the mediating variable (attitudes of people), and the relationship between the mediating variable and the outcome variable, commitment to stay home. It was observed that alternative narratives on COVID-19 and media coverage of COVID-19 were significant on the attitude of people. The message on COVID-19 did not significantly influence the attitude of people. The attitude of people had a significant relationship with the commitment level of people to stay home. This implied that H2–H4 were accepted whilst H1 was rejected.

**TABLE 4:** Structural analysis of the study.

Study’s hypothesis	Hypothesis	Path coefficients	*T*-statistics	*p*	Supported/Rejected
MIC AT	H1	0.067	0.572	0.568	Rejected
MV AT	H2	0.297	3.080	0.002 ***	Supported
AN AT	H3	0.327	3.043	0.003 ***	Supported
AT C	H4	0.762	22.182	0.000 ***	Supported

Note: AN, alternative narratives; AT, attitudes; C, commitment to stay home; MIC, message; MV, media coverage; *p* < 0.05.

*** , *p*-value is less than 0.001;

**, *p*-value is less than 0.01; *, *p*-value is less than 0.05.

### Goodness-of-fit assessment

The goodness-of-fit (GOF) statistics was assessed using a procedure by Tenenhaus et al. (2005), where the averages of the AVE were first multiplied by the averages of the R² value, after which the multiplied value was squared to determine the model fit.


$$\begin{array}{l} \text{GOF}=\surd \text{AVE}\times {\text{R}}^{2}\\ \;=\surd 2.043\times 0.602\\ \;=\surd 1.229886\\ \;=1.10 \end{array}$$

The calculated global GOF was 1.10. It can be concluded that the model had a superior fit because this statistic surpassed the threshold of GOF > 0.36 as recommended by Wetzels, Odekerken-Schröder and Van Oppen (2009).

Table 5 presents the profile of the study sample. It was observed that 53% of the participants were women and 34% of the participants were between 26 and 35 years old, followed by 35 years and above, which recorded 26%. A majority of the respondents (63%) were married, whilst respondents with ‘first degree’ as their highest qualification were the most, representing 32%.

**TABLE 5:** Demographic characteristics of the respondents (*N* = 352).

Variable	Description	Cumulative	Percentage
Gender	Male	167	47
	Female	185	53
	Total	352	100
Age	Below 18 years	61	17
	18–25 years	77	23
	26–35 years	121	34
	Plus 35 years	93	26
	Total	352	100
Marital status	Single	92	26
	Married	221	63
	Divorced	39	11
	Total	352	100
Educational background	MSLC or JHS	15	4
	SSCE	95	27
	Diploma	42	12
	Degree	111	32
	Postgraduate	89	25
	Total	352	100

MSLC, Middle School Leaving Certificate; JHS, Junior High School; SSCE, Senior School Certificate Examination.

## Discussion

This study was conducted to determine the relationship between the message, media coverage and alternative narratives on the attitudes of people in the Kumasi Metropolis during the COVID-19 pandemic. It examined the relationship between the attitudes of people and their commitment to stay home during the pandemic period. The analysis showed that alternative narratives were strongly related to attitudes, and media coverage had a weaker coefficient. These findings are consistent with Raghubir (2008), who observed from a high health risk research stream that information rate has a greater likelihood to influence the perception of health risk. It shows that adopting diverse strategies could have had a greater influence on the attitude of people to stay home during the COVID-19 period, as revealed by the respondents through their comments in the open-ended section of the questionnaire. An alternative narrative of a crisis event describes an account of procedures that discards the principal narrative (Nied et al. 2017). The alternative narratives, such as involving public health-related personnel in spearheading the activities during the COVID-19 campaigns, would have affected the attitude of people regarding the information they would have received. Nyarko, Serwornoo and Azanu (2021) revealed how politicians in Ghana used the COVID-19 in diverting attention to their political activities instead of focusing on the fight against the pandemic. The findings also show that media coverage significantly influenced the attitudes of people, but its impact was not very strong, whilst the message on COVID-19 was very weak in terms of its influence on the attitude of people to stay home during the pandemic period. According to Stremersch (2008), people or consumers face a high level of doubt and imperfect information in a context in which wrong decisions have an important impact on their well-being.

Farooq, Laato and Islam (2020) observed that the persistent provision of information by different people contributed to the information overload and overconcern amongst people during the pandemic. Using the government communicators and government-appointed health officials in spearheading the campaign weakened the message that was communicated to people. It was observed that people regarded most of the campaign activities as an opportunity for the government to make a good impression on the international community. Political opponents also created countermeasures in refuting the claims of the government in the fight against the virus. This, amongst other factors, affected the content of the actual message. Sarkhel et al. (2020) posited that the increase in the consumption of news led to an increase in people avoiding the news during the COVID period. Relying on the outcome of the study, the findings have shown that non-partisan experts or health professionals from various health sectors in the country ought to have been used during the entire campaign process and the various associations under Ghana Health Service would have been more appropriate in sending the right messages to the people. The overwhelming effect of the COVID-19 was attributed to the information overload and ‘infodemic’ (Rathore & Farooq 2020).

Finally, it was also observed that the attitude of people influenced their commitment to stay at home. According to Mohammed et al. (2021), valuable information could become a help if it is handled well. It was evident from the study that people were willing to stay home as long as the campaign was free from indirect political inclinations, structured messages and other televised information on affected persons. The findings are consistent with Zavattaro and McCandless (2020) who observed that others respond well to actual solutions.

## Implication for theory and practice

This study contributes to the literature on the relevant dimensions of health communication campaigns. Most governments, groups or individuals employed different approaches in minimising the spread of the coronavirus. Signalling theory provides a way by which signals from independent experts could help convey messages to people. Whilst the traditional operationalisation of the campaign was planned mainly by governments in Africa, this study has found it to be an insignificant component in behaviour modification towards compliance. Involving professional health officials in leading health campaigns has a greater likelihood of changing people’s behaviour. While most previous studies on the coronavirus were conducted in the Western world, limited studies have addressed issues within the sub-Saharan African context, and this study with humanities and business research perspectives fills the gap. This research provides empirical evidence from Ghana, a developing economy. The study has some managerial implications for building a resilient health communication strategy that could improve the compliance level of targeted respondents. The need for health authorities to be given the full responsibility by governments to have campaign blueprints and train personnel to convey definite information cannot be overemphasised. Finally, alternative narratives were also found to be the greatest predictor of the attitude of the people. Strong management and linkage with popular celebrities could be used by the health officials to create strong remembrances and images for people to adhere to directives associated with campaigns. Policy makers ought to pursue these aims as part of their campaign-building compliance activities.

## Conclusion

In this article, the authors investigated the effect of the content of the message, media coverage and alternative narrative interventions on the attitude and commitment levels of people to stay at home during the COVID-19 pandemic in Ghana. The results showed that the content of the message did not correlate with the attitude of Ghanaians, whilst the alternative narratives and media coverage had a strong and positive correlation with attitude. Also, the attitude of people significantly influenced their commitment to stay home. It was evident that the message by the government during the COVID-19 period insignificantly influenced the attitude of people. The involvement of public health officials was a good signal and would have greatly influenced people’s attitude and commitment to stay at home during the pandemic period. The authors, therefore, conclude that developing countries in Africa ought to fight health-related pandemics using a non-partisan health directorate and officials rather than government-appointed health experts or government officials in spearheading the activities in such situations.

## Limitations of the study and directions for future study

This study had some limitations. First and foremost, the study employed a non-probability sampling technique. Hence, the findings of the study cannot be generalised to represent the population of the study area. Secondly, the study focused on participants within the Kumasi Metropolis. Future studies should include respondents from other regions of Ghana. Finally, there is the need for an exploratory study to be conducted on two different countries in the subregion – regarding the use of government officials and neutral health professionals in the quest to control probable pandemics like the COVID-19.

## Appendix 1: Measurement items

### Commitment

1. I feel emotionally attached to staying home.
2. It would be hard for me not to stay home.
3. I would choose to stay home because I have a sense of responsibility for it.

### Messages on the COVID-19

1. Messages on the COVID-19 focused on the problem.
2. Messages on the COVID-19 focused on other activities.

### Media coverage on the COVID-19

1. I am comfortable with the media coverage of the pandemic.
2. I am pleased with the overall media-related activities associated with the COVID-19 campaigns.

### Alternative narratives

1. If I need to change things on the COVID-19 campaigns, there are other things I would consider.
2. I might be happy with a different campaign strategy.
3. There are other campaign approaches that would have probably been more satisfying than the current one.
4. Compared to this campaign, there are other persons I would have considered leading.

### Attitude

1. I am naturally moved to comply with the COVID-19 campaign activities.
2. Adhering to the general rules on the COVID-19 campaign was a better choice for me.
3. I was confident in observing the rules associated with the COVID-19 campaigns.
